# Microcavity Enhanced Raman Spectroscopy of Fullerene C_60_ Bucky Balls

**DOI:** 10.3390/s20051470

**Published:** 2020-03-07

**Authors:** Vinayaka H. Damle, Miri Sinwani, Hagit Aviv, Yaakov R. Tischler

**Affiliations:** Department of Chemistry, Institute for Nanotechnology and Advanced Materials (BINA), Bar-Ilan University, Ramat Gan 52900002, Israel; damlevi@biu.ac.il (V.H.D.); mirisin.85@gmail.com (M.S.); hagit.aviv@biu.ac.il (H.A.)

**Keywords:** CERS, fullerene C_60_, DFT

## Abstract

Raman spectroscopy is a widely used characterization technique in material science. It is a non-destructive tool with relatively simple instrumentation, and provides intrinsic qualitative information of analytes by probing their vibrational modes. In many cases, Raman enhancement is essential for detecting low-intensity signals in high-noise environments, spectrally unresolved features, and hidden modes. Here we present optical and Raman spectroscopic characterization of fullerene C${}_{60}$ in a gold microcavity. The fabrication of single-layered gold mirrors is facile, low cost and direct but was proven to give considerably significant enhancement. The findings of this work demonstrate the cavity resonance as a powerful tool in obtaining tunability over individual peak for selective enhancement in the tuned spectral range. The PL of the material within the cavity has demonstrated a red shift assumed to be caused by the low-energy transitions. These transitions are induced by virtual low-energy states generated by the cavity. We further observe that adopting this principle enables resolution of active Raman modes that until now were unobserved. Finally, we assigned the new experimentally observed modes to the corresponding motions calculated by DFT.

## 1. Introduction

Raman spectroscopy is a powerful spectroscopic tool in understanding the bonds, mechanics and dynamics of molecules in addition to surface stress or strain [1,2,3,4]. However, recording and resolving these spectral signatures is a strenuous task due to relatively low signal to noise ratio (SNR). The low SNR is due to lower Raman scattering cross sections and sometimes different intra or inter molecular quenching phenomenon [5]. Therefore, developing different methods to enhance and resolve these fine low-intensity spectral features is more rewarding as well as challenging for the scientific community. In this direction a lot of work has been done in developing different techniques to enhance Raman peak intensity which include surface engineering; such as using metal nanoparticles or thin metallic layers above or beneath the measured material. Among those techniques are Surfaced Enhanced Raman Spectroscopy (SERS), Plasmon Enhanced Raman spectroscopy (PERS), and Tip Enhanced Raman spectroscopy (TERS) [6,7,8]. The field of plasmon enhanced Raman spectroscopy (PERS) evolved along the discovery of various plasmon active materials. It is well established that SERS can be fundamentally achieved by chemical enhancement or electromagnetic enhancement. Earlier reports indicate that electromagnetic enhancement can be achieved by near field enhancement and/or radiation enhancement [9]. Cavity Enhanced Raman Spectroscopy (CERS) was developed over the last few decades [10,11]. In this method, planar optical micro-cavities are fabricated as resonators. These resonators trap light in a specific spectral range centered around the resonant wavelength $\lambda_{c}$. The relation between the resonating wavelength $\lambda_{c}$ and the spacing between the two parallel mirrors is given by

(1) $$d=m\lambda_{c}/2n$$

where ‘*n*’ is the refractive index (RI) of the medium in between the mirrors and ‘*m*’ is a material specific constant. The constructive interference of the standing waves within the cavity enhances the amplitude at the resonant frequency of the cavity. The high reflection of the mirrors traps light inside the cavity leading to higher Raman interaction cross-section, thus the little light that escapes the cavity, carries strongly enhanced Raman signals that overcome the losses of the trapped light. Varying the ‘*d*’ allows tunability of the cavity to obtain the required resonant mode in the cavity as reported earlier [12]. Such tunability enables selective probing of the analyte for different light matter interactions induced or enhanced by the cavity resonance, and enables observation of unresolved Raman peaks.

C${}_{60}$ is an important organic n-type semiconductor with low activation energy of 0.1–0.3 eV, the conductivity within the material is attributed to intrinsic oxygen-related defects [13]. Earlier works on vibrational spectroscopy of C${}_{60}$ mainly focused on Raman and Infrared spectroscopies [14]. This includes theoretical work on symmetry analysis, developing models to understand isotopes, solid state effects as well as normal mode calculations using DFT within local density approximations [15]. The theoretical work has provided a good basis to understand the C${}_{60}$ molecule. However, all the spectroscopic signatures claimed by the theoretical reports were barely distinguishable due to very low SNR [16,17,18]. Furthermore, many efforts were made combining Raman, Infrared, and Neutron scattering spectroscopies towards resolving and understanding the fine structure of Raman peaks [19,20]. SERS of C${}_{60}$ on noble-metal surfaces was first reported in 1992 [21,22]. Raman frequencies of C${}_{60}$ on Cu and Ag surfaces were reported to induce shifts of as high as 23 to 28 cm${}^{-1}$. It was reported that these shifts were a result of surface interactions due to charge transfer in the surfaces with respect to certain vibrational modes and symmetries [23]. Heretofore, a lot of effort is put on enhancing and resolving Raman peaks of C${}_{60}$ thin films, by using surface enhancement techniques which include SERS and TERS [24,25,26]. These studies have consistently reported surface induced shifts in Raman peaks due to surface interactions mainly with the π electrons of C${}_{60}$. Furthermore, an anomalous shift in Raman peak was explained by the possibility of polymerization of C${}_{60}$ molecules [27].

In this paper, we present an investigation of a microcavity enhanced Raman spectrum of Fullerene C${}_{60}$ using cavity resonance in between two gold mirrors. The Fabrication of single-layered gold mirrors is facile, low cost and direct but was proven to give considerably significant enhancement. This technique enabled us to experimentally observe a Raman peak that was only theoretically resolved and for other peaks, a significant enhancement was witnessed. The theoretical calculations were performed by the density functional theory (DFT) and the resonant modes interacting with cavity resonance were identified. In addition, we demonstrate a selective enhancement of the C${}_{60}$ Raman peaks by tuning the cavity resonance through variation of the distance between the mirrors.

## 2. Materials and Methods

### 2.1. Materials

The following analytical-grade chemicals were purchased from Merck and used without further purification: Micro-90 semiconductor grade detergent, acetone (99.9%), isopropanol (99.5%), polystyrene (PS, average Mw: 400,000). Fullerene C${}_{60}$ (99.9%) was purchased from Arcos organics, New Jersey, USA. Gold granules (99.999%) was purchased from Holland- Moran, Israel. Deionized (DI) water was obtained by purifying water through a Barnstead EASY Pure II osmosis system (Thermo Fisher Scientific Inc. Israel).

### 2.2. Methods

#### 2.2.1. Cavity Preparation

Glass substrates were cleaned via ultra-sonication in Micro-90 solution followed by deionized distilled water, acetone and isopropanol. Thin films of C${}_{60}$ was sandwiched between uniform thin films of gold of about 30 nm thickness using thermal vapor deposition (Kurt J. Lesker Nano36 at a base pressure maintained around 10${}^{-6}$ Torr). Deposition rate of gold was set to 0.2 Å/s while for C${}_{60}$ as it being organic, care was taken in deposition and a constant deposition rate of 0.1 Å/s was set. The rate was monitored using pre-calibrated crystal monitors placed inside the vacuum chamber, and varied by changing the current flowing through the Tungsten boats that hold the materials. The current rises the boat’s temperature which in turn leads to evaporation of the materials. The C${}_{60}$ thickness varied from 95 nm, 105 nm, 115 nm, 125 nm, 135 nm, 145 nm to 160 nm. No additional spacer was used to avoid RI inhomogeneity inside the cavity; however, the thickness differences outside a cavity did not reveal spectral changes. Reference cavities were prepared using optically transparent spin coated PS of similar range of thickness as mentioned above and was sandwiched between gold layer.

#### 2.2.2. Metrological Characterization

Thickness measurements were carried out using Veeco make, Dektak150 profilometer and Zeiss 982 HR-SEM. As per the requirements of HR-SEM analysis special samples were prepared on silicon substrate.

#### 2.2.3. Photo-Luminescence and Raman Spectra

Photo-luminescence (PL) and co-located Raman spectra were measured in a dual laser confocal micro-Raman/PL system (LabRam HR) with the aid of automated laser and filter switching that enabled stable and accurate measurements. For the Raman characterization, we used excitation wavelengths of 532 nm and 784 nm. The power of the laser was adjusted to 3 mW, and the integration time was 120 s to detect the weak Raman signals. For the PL characterization we used an excitation wavelength of 532 nm and adjusted the laser power to 5 μW for integration time of 1 s. In each measurement, an excitation laser beam was first transmitted through an objective of either 50X (N.A. = 0.75) or 100X (N.A. = 0.9) to a certain spot on the material surface, then the scattered light was collected by the same objective and directed to the spectrometer portion of the instrument. The grating grove density was 600 g/mm across the measurements. Raman frequencies were calibrated using a silicon wafer and all the experiments were conducted at room temperature.

#### 2.2.4. Transmission Spectroscopy

Transmission measurements were carried out on cavities containing C${}_{60}$ and on reference cavities containing PS of the same thickness as that of C${}_{60}$, using Cary Eclipse fluorescence spectrophotometer in a steady-state mode.

#### 2.2.5. DFT Calculations

DFT calculations used the hybrid-functional $B_{3}LYP$ with the exchange-correlation that combines the respective established mathematical models with certain coefficient for each expression [28,29]. Specifically, the functional use the Becke functional with 3 parameters for exchange, and it also includes the correlation functional of Lee, Yang, and Parr (LYP) [30]. The B3LYP functional was chosen since it produces reasonable accurate calculations with low computational resources. The calculations of the normal vibrational modes were executed with the basis sets of 3 - 21 + G* and 6 - 31 + G* for neutral C${}_{60}$ in a singlet state. The details are discussed in Supplementary Information.

## 3. Results and Discussion

Out of 174 theoretically predicted possible modes of vibration, 46 fundamental modes were previously reported. The symmetries and number of modes reported for each symmetry are listed in Table 1 [17]. Of these 46, the Ag and Hg modes are reported to be Raman active (bolded). This work focuses on selective CERS of Hg, Ag1, and Ag2 vibrational modes.

**Figure 1:**
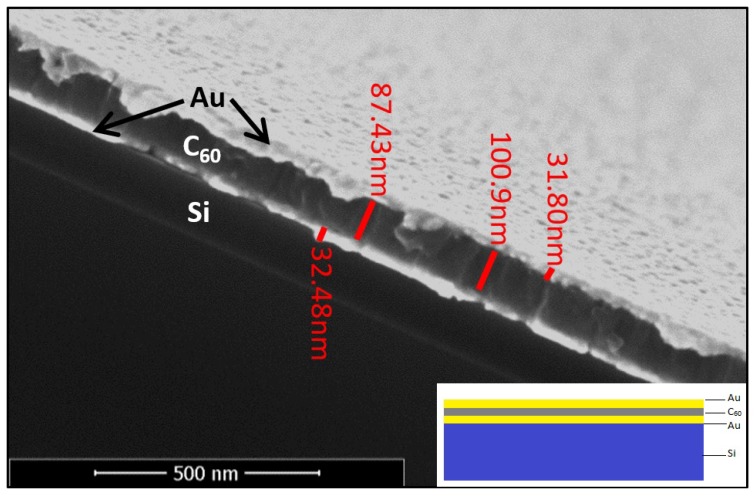
HR-SEM micrograph of C${}_{60}$ in Au micro cavity, inset schematic cross-section of the cavity.

Metrological characterization was carried out using HR-SEM for reconfirming the different films’ thickness, recalibrate the deposition protocols, and verify the coatings’ uniformity. The cross-section micrograph, presented in Figure 1, indicate that the dimensions of the layers are uniform with the coating parameters and are within the limits of instrumental error. It is also observed from the micrograph that the layers are smooth and continuous.

**Figure 2:**
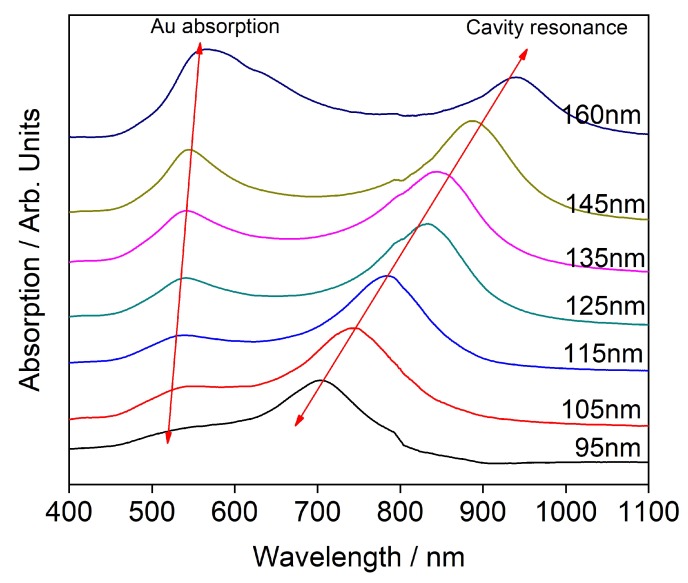
Absorption spectrum of C${}_{60}$ in gold cavity.

**Figure 3:**
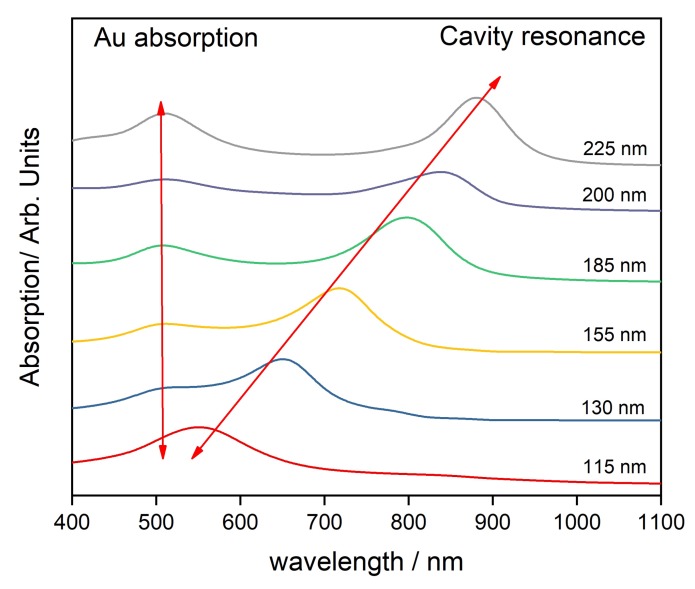
Absorption spectrum of PS in gold cavity.

**Figure 4:**
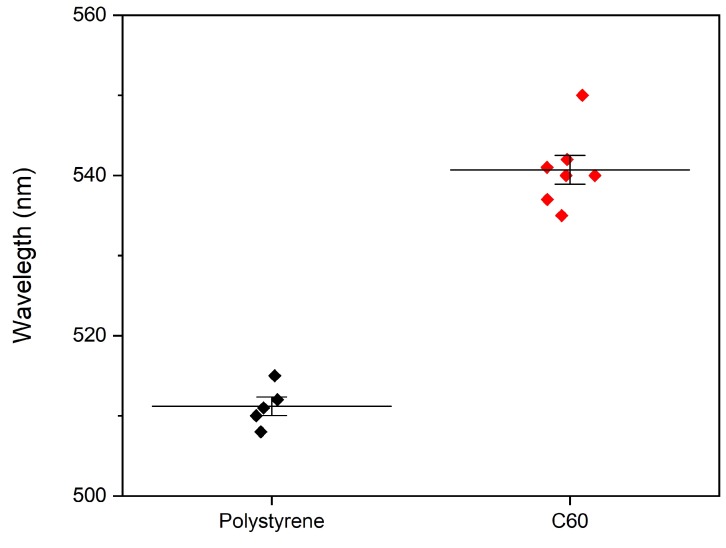
Comparison of Au SPR peaks in PS and C${}_{60}$ cavities.

**Figure 5:**
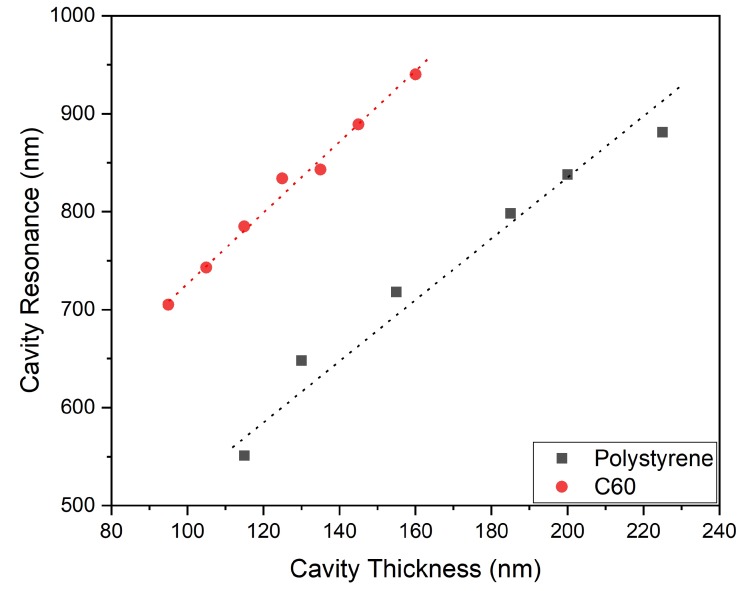
Comparison of cavity resonance with variation of cavity thickness of C${}_{60}$ and PS cavities.

Gold thin films were observed to transform from islets to a continuous film at above 25 nm thickness in earlier work of our group [31]. The continuous film demonstrated minimal SERS enhancement and in order to study the CERS phenomenon with minimal contribution from surface enhancement, we chose our gold films thickness to be 30 nm. The absorption spectroscopy data presented in Figure 2 indicate two peaks. One of them around 500 nm and the other at a higher wavelength. It is observed that the second peak originates from cavity resonance and this was confirmed by carrying absorption spectroscopy of the reference cavities of comparable cavity thickness using PS spacing layer as observed from Figure 3. The difference in the position of the cavity resonance peaks between C_60_ and PS occurs because the cavity resonance is largely dependent on the spacing between the reflecting mirrors and the RI of the material within the cavity. In case of Figure 2 and Figure 3, the C${}_{60}$ has RI of around 2.18 whereas the PS is a more transparent material with RI of 1.5. The absorption spectrum of 30 nm thick gold film presented in Figure S2 indicates an absorption maximum around 500 nm in agreement with literature [32]. It is well known that the gold’s absorption peak evolves due to Surface Plasmon Resonance (SPR) [33]. The SPR depends on local environment, and when the material inside the cavity is replaced, we observe a shift in the absorption peak. This shift is presented in Figure 4. Figure 2 and Figure 3 demonstrate a secondary absorption maximum in both analyte and reference cavities to the absorption maximum of gold. It is further clear that the cavity resonance shows a red shift in the resonating wavelength with the increase in layer’s thickness as indicated by Figure 5. This red shift is a result of the change in the trapped wavelength inside the cavity, while for the thin film, the absorption peak wavelength is constant as there is no wavelength trapping. The linearity of the red shift as indicated by Figure 5 further supports our assignment as resonance shifts occurring from the distances between the mirrors are linear as expected from Equation (1). In addition, the slope of the shift varies with the material sandwiched inside the cavity. This slope is determined by the RI of the material which is discussed in detail elsewhere [12]. Figure 5 presents the different slopes of PS and C${}_{60}$ when sandwiched inside the cavity.

**Figure 6:**
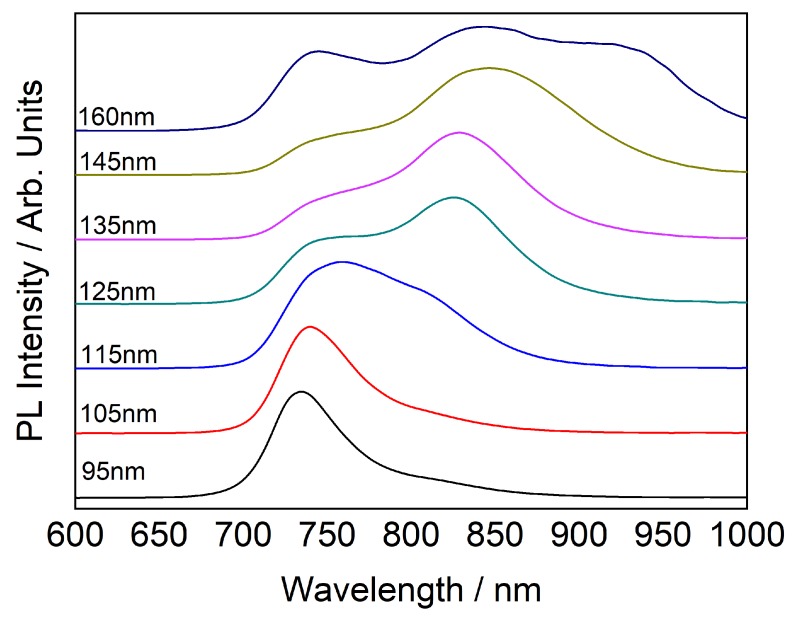
Evolution of PL spectrum with cavity thickness.

PL measurements indicates emergence of new secondary and tertiary PL peaks with increase in cavity thickness, these peaks consistently show considerable red shift as shown in Figure 6. The emergence of secondary PL peak at higher wavelength is assigned to a lower band gap transition that is generated from placing the C${}_{60}$ inside a cavity, for relatively thin films, the mirrors are too close to enable the co-existence of lower energy bands, therefore we observe this transition only in 160 nm thick layer. The tertiary PL peak is assigned to the original cavity resonance. These low bandgap transitions are reported for similar structures such as quantum dots [34]. A possible explanation for the lower bandgap transition is that surface enhanced PL for C${}_{60}$ with Au particles is achieved by exciting the surface plasmons [35]. Such excitations bring in very strong local fields and when the cavity resonance matches the energy of excitation photon, the transition probabilities of stimulated optical processes increase drastically. Specifically, for C${}_{60}$, such non-linearities were reported elsewhere [36].

**Figure 7:**
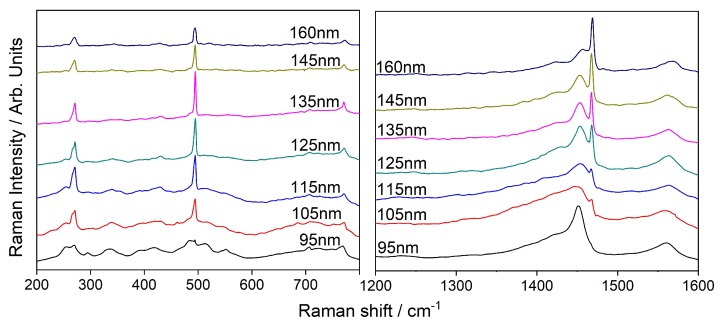
Raman spectrum of C${}_{60}$ in gold cavity excited with 784 nm.

CERS was carried out using both 784 and 532 nm laser excitations, the spectra are presented in Figure 7. Both wavelengths were chosen since previous studies have shown SERS spectra diversity for these laser excitations [31]. In this work we primarily concentrate on the shifts at 270 cm${}^{-1}$, 495 cm${}^{-1}$, and 1468 cm${}^{-1}$. Peaks obtained with 784 nm laser excitation shown in Figure 7 are in agreement with the fundamental vibrational modes associated with the symmetry groups Hg, Ag1, and Ag2 found in the literature [37]. Figure 7 also indicates that the spectral region that undergoes enhancement is relative to the distance between the mirrors. When the distance is 95 nm, the maximum enhancement is at 1452 cm${}^{-1}$, while bigger distances lead to a red shift in the enhanced spectral region. This trend is expected from the equation of cavity resonance discussed in the introduction section. This tunability enabled us to resolve the Raman active mode at 1468 cm${}^{-1}$ by selective enhancement. Variation in signal intensity of two specific peaks along this tuned spectral range, at 1452 and 1468 cm${}^{-1}$, is presented in Figure S4, the plots show that varying the cavity thickness leads to selective Raman enhancement.

**Figure 8:**
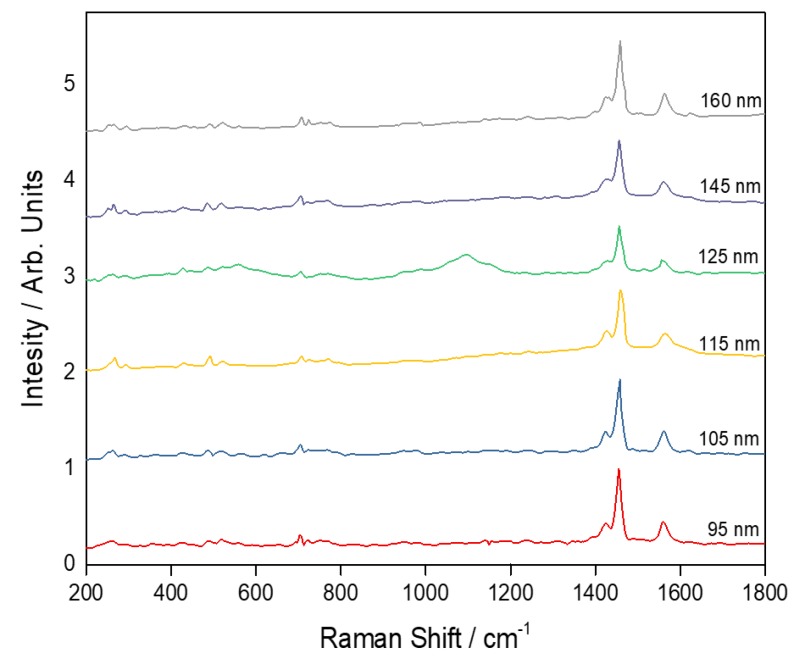
Raman spectrum of C${}_{60}$ in gold cavity excited with 532 nm.

Further proof of the importance of pre-calculating the cavity resonance before designing the experiment to avail the expected enhancement is found in Figure 8. Unlike excitation with 784 nm, Raman studies on the same samples with 532 nm excitation do not enhance or resolve the Raman peak at 270 cm${}^{-1}$ and they reveal a pronounced Raman peak at 1468 cm${}^{-1}$ without enhancement or a red shift effect. Since the fabrication was designed to generate enhancement for 784 nm excitation, the absence of enhancement in the spectrum obtained from 532 nm excitation is expected. Figure S3 presents the changes in intensity of the different Raman peaks across the spectrum and their calculated enhancement factor. In addition to expected changes in the enhanced spectral region with the thickness variation, it is clear that some peaks in certain thicknesses cannot be resolved, for example the Raman peak at 1468 cm${}^{-1}$ is not resolved in 95 nm layer’s thickness.

**Figure 9:**
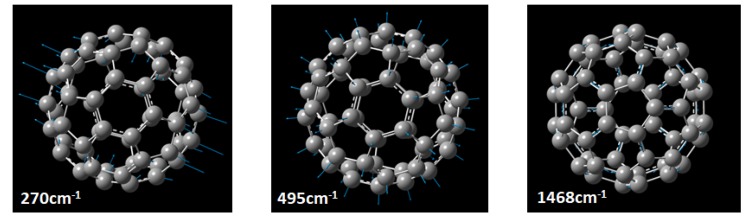
Graphical representation of Wagging, Breathing and Twisting vibrational modes of C${}_{60}$ molecule.

DFT Calculations carried out for individual C${}_{60}$ molecule show that the shifts observed at 270 cm${}^{-1}$, 495 cm${}^{-1}$, and 1468 cm${}^{-1}$ are assigned to Wagging, Breathing and Twisting vibrational modes of C${}_{60}$ molecule, respectively as presented in Figure 9. Further details of the DFT calculations are discussed in detail and presented through Figure S1 and Tables S1–S3 in the Supplementary Information. The experimental spectra are consistent with the theoretically predicted modes. The enhancement method we presented, not only increased the intensity of previously detected Raman shifts, but also revealed new active modes that until now were found only in theoretical calculations.

## 4. Conclusions

In this paper, we demonstrate an investigation of CERS of Fullerene C${}_{60}$. This technique enabled us to experimentally observe new Raman peaks that we theoretically predicted. We used DFT calculations to assign the Raman peaks to the correlated vibrational modes. In addition, we show a selective enhancement of the C${}_{60}$ Raman peaks by varying the cavity distance. We believe this technique when carefully engineered, along with theoretical calculations, can be used to characterize materials for their weak or yet to be revealed active modes.

## Figures and Tables

**Table 1:** The fundamental vibrational modes of C${}_{60}$. Highlighted modes are Raman active.

Symmetry	Ag	Au	T1g	T1u	T3u	T3g	Gg	Gu	Hg	Hu
**Number of Modes**	**2**	1	3	4	5	4	6	6	**8**	7

## Supplementary Materials

The following are available at https://www.mdpi.com/1424-8220/20/5/1470/s1.

## Abbreviations

The following abbreviations are used in this manuscript:

PL	Photo-Luminescence
DFT	Density Functional Theory
SNR	Signal to Noise Ratio
SERS	Surfaced Enhanced Raman Spectroscopy
PERS	Plasmon Enhanced Raman Spectroscopy
TERS	Tip Enhanced Raman Spectroscopy
CERS	Cavity Enhanced Raman Spectroscopy
PS	Polystyrene
DI	Deionized
NA	Numerical Aperture
