# Supplementary material for: Microcavity Enhanced Raman Spectroscopy of Fullerene C60 Bucky Balls
Source: Sensors (Basel). 2020 Mar 7;20(5):1470. doi: 10.3390/s20051470 (PMC7085650; doi:10.3390/s20051470)
Supplement: Supplementary file 1 [file sensors-20-01470-s001.zip › sensors-711738-supplementary-done/sensors-711738-Supplymentary/Definitions/logo-mdpi-eps-converted-to.pdf]

MDPI
