# Supplementary material for: Microcavity Enhanced Raman Spectroscopy of Fullerene C60 Bucky Balls
Source: Sensors (Basel). 2020 Mar 7;20(5):1470. doi: 10.3390/s20051470 (PMC7085650; doi:10.3390/s20051470)
Supplement: Supplementary file 1 [file sensors-20-01470-s001.zip › sensors-711738-supplementary-done/sensors-711738-Supplymentary/sensors-711738-supplementary.pdf]

sensors

MDPI

Article

Microcavity Enhanced Raman Spectroscopy of Fullerene C_{60} Bucky Balls

Supplementary Materials: The calculations of the normal vibrational modes were performed with the basis sets of 3- 21+G* and 6-31+G* for neutral C_{60} in a singlet state. As mentioned earlier, C60 contains 174 normal modes which are reduced to total number of 46 distinct normal modes, because many modes are degenerate. Calculating vibrational modes of neutral singlet state C_{60} with a basis set of 3-21+G* revealed 16 negative normal modes as listed in Table S1.

Table S1. Negative vibrational modes calculated with basis set of 3-21+G*

Mode	Frequency (cm^{-1})	Mode	Frequency (cm^{-1})
1	-1008.12	9	-476.11
2	-1004.93	10	-467.13
3	-1004.35	11	-459.99
4	-1001.39	12	-430.33
5	-619.07	13	-428.72
6	-616.93	14	-426.58
7	-615.26	15	-422.56
8	-484.25	16	-417.98

We concluded that using a small basis set of 3-21+G* is not sufficient to calculate the vibrational modes of our system. Using the basis set of 6-31+G* revealed 174 normal modes without any negative vibrational modes. To obtain more accurate values of the frequencies, we multiplied the calculated normal modes with scaling factor of 0.98. To visualize the degeneration of the calculated normal modes, we plotted the normal modes versus their calculated frequency as shown in Figure S1. We extracted from the degeneracy plot 45 normal modes, listed in Table S2, and we believe the normal modes at 724-728 cm-1 can be divided to two and assigning these two modes requires further investigation.

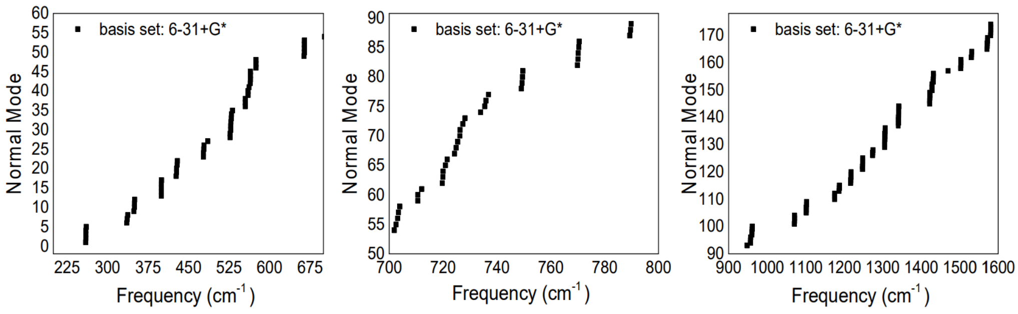

Figure S1. Illustration of the calculated normal modes of neutral C_{60} in at singlet state versus the modes' frequency.

Table S2. Degenerate normal modes of neutral C_{60} in a singlet state

Mode	Degenerate modes(cm^{-1})	Mode	Degenerate modes(cm^{-1})	Mode	Degenerate modes(cm^{-1})
1	260	16	710	31	1187
2	336	17	720	32	1217
3	350	18	724-728	33	1248
4	400	19	733	34	1274
5	428	20	735	35	1306
6	478	21	749	36	1340
7	485	22	770	37	1342
8	528	23	789	38	1422
9	531	24	824	39	1428
10	555	25	948	40	1431
11	562	26	957	41	1470
12	565	27	960	42	1503
13	575	28	1071	43	1531
14	664	29	1102	44	1571
15	703	30	1175	45	1581

We further investigated the correlation between the computed 10 Raman active normal modes of a singlet state C_{60} to the experimental Raman active modes obtained in the SERS spectra of C_{60} excited with laser excitations of 532 nm and 784 nm. Table S3. shows that the theoretical normal modes of C_{60} in a singlet state nicely fit to the experimental Raman modes excited with wavelength of 784 nm, whereas excitation wavelength of 532 nm does not reveal two calculated Raman modes in addition to significant downshifting of the pentagonal pinch mode.

Table S3. Comparison between calculated Raman modes in singlet state C_{60} to experimental Raman modes obtained i

Mode	DFT	532 nm	Deviation	784 nm	Deviation
1	259.8666	263	4.1334	265	5.1334
2	427.8876	428	0.1124	425	-2.8876
3	485.7174	490	4.2826	490	4.2826
4	703.1794	723	19.8206	707	3.8206
5	769.9272	772	2.0728	769	-0.9272
6	1102.7450	-	-	1098	-4.745
7	1248.6570	-	-	1247	-1.6572
8	1422.6660	1421	-1.6660	1421.84	-0.8260
9	1470.3720	1458	-12.3724	1466	-4.3724
10	1581.2790	1561	-20.2790	1565.83	-15.4490

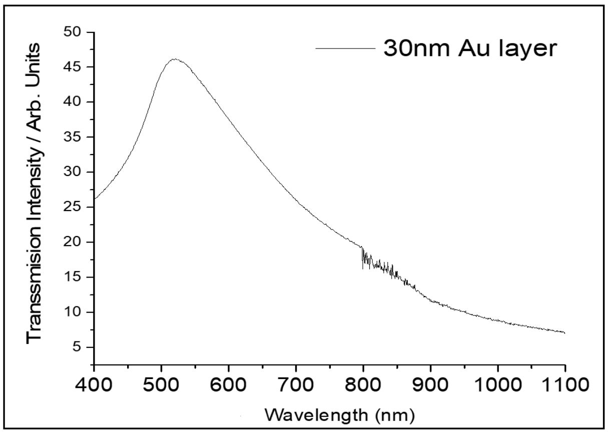

Figure S2. Transmission spectrum of 30nm thick gold

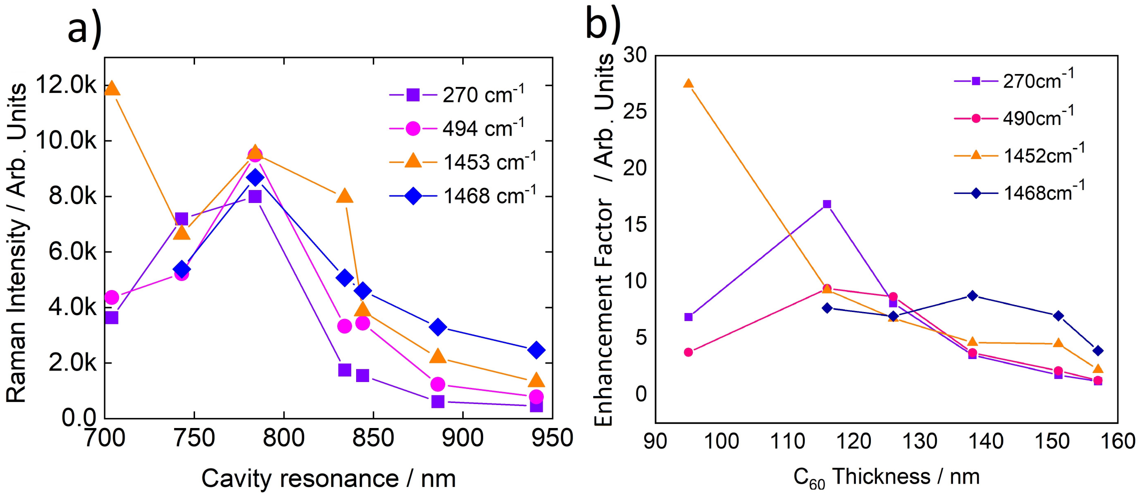

Figure S3 a) Graphical illustration of intensity variation across different Raman peaks of C_{60} as compared with the cav

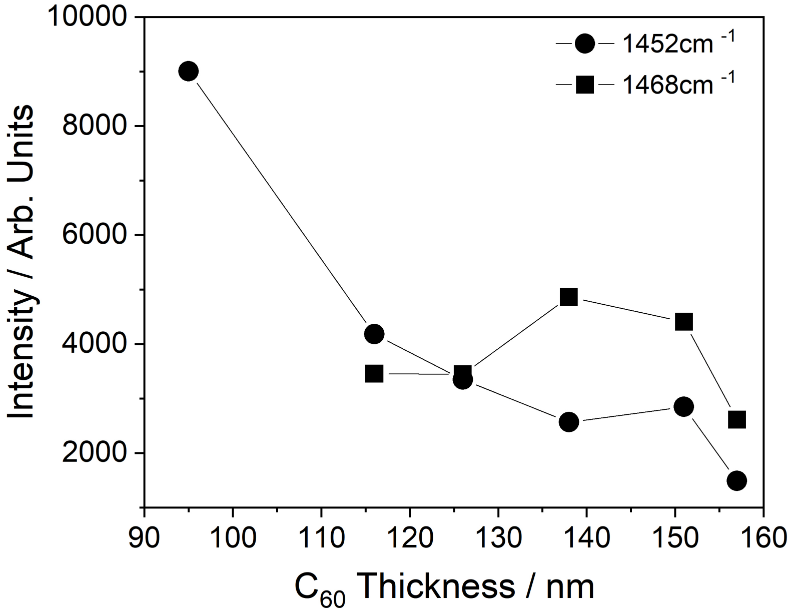

Figure S4 Variation in signal intensity variation across selectively enhanced Raman peaks of C_{60}

© by the authors. Licensee MDPI, Basel, Switzerland. This article is an open access article distributed under the terms and conditions of the Creative Commons Attribution (CC BY) license (http://creativecommons.org/licenses/by/4.0/).
