# Supplementary figures and images for: Microcavity Enhanced Raman Spectroscopy of Fullerene C60 Bucky Balls
Source: Sensors (Basel). 2020 Mar 7;20(5):1470. doi: 10.3390/s20051470 (PMC7085650; doi:10.3390/s20051470)

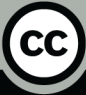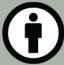

BY

Supplement: Supplementary file 1 [file sensors-20-01470-s001.zip › sensors-711738-supplementary-done/sensors-711738-Supplymentary/Definitions/logo-ccby-eps-converted-to.pdf]

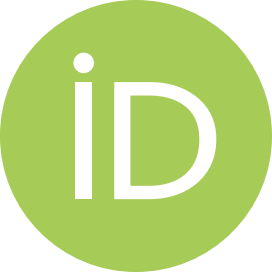

Supplement: Supplementary file 1 [file sensors-20-01470-s001.zip › sensors-711738-supplementary-done/sensors-711738-Supplymentary/Definitions/logo-orcid-eps-converted-to.pdf]

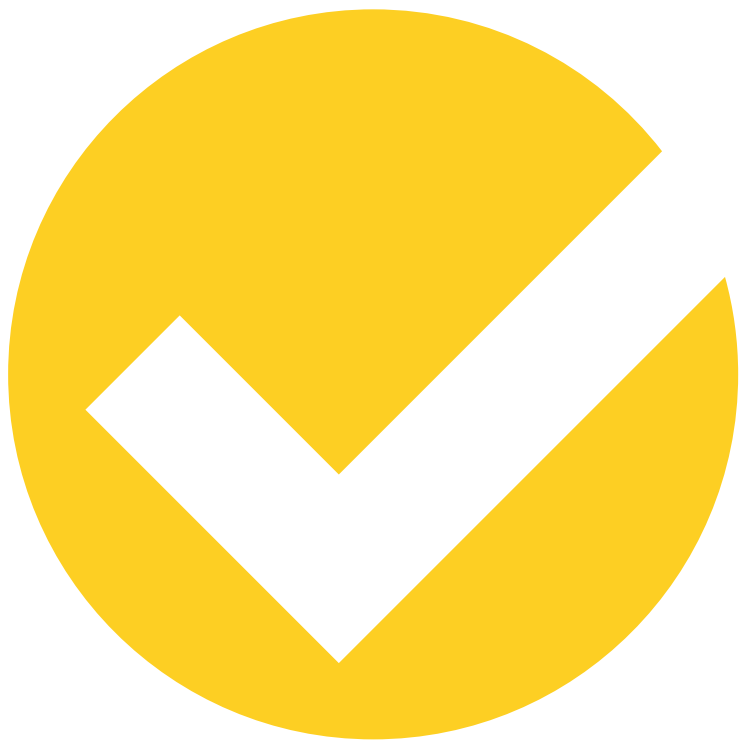

check for  
updates

Supplement: Supplementary file 1 [file sensors-20-01470-s001.zip › sensors-711738-supplementary-done/sensors-711738-Supplymentary/Definitions/logo-updates.pdf]

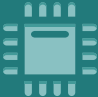

*sensors*

Supplement: Supplementary file 1 [file sensors-20-01470-s001.zip › sensors-711738-supplementary-done/sensors-711738-Supplymentary/Definitions/sensors-logo-eps-converted-to.pdf]

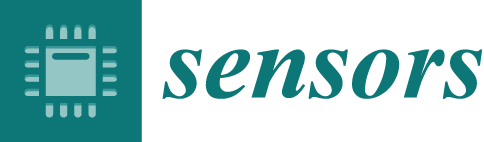

Supplement: Supplementary file 1 [file sensors-20-01470-s001.zip › sensors-711738-supplementary-done/sensors-711738-Supplymentary/Definitions/sensors-logo.png]

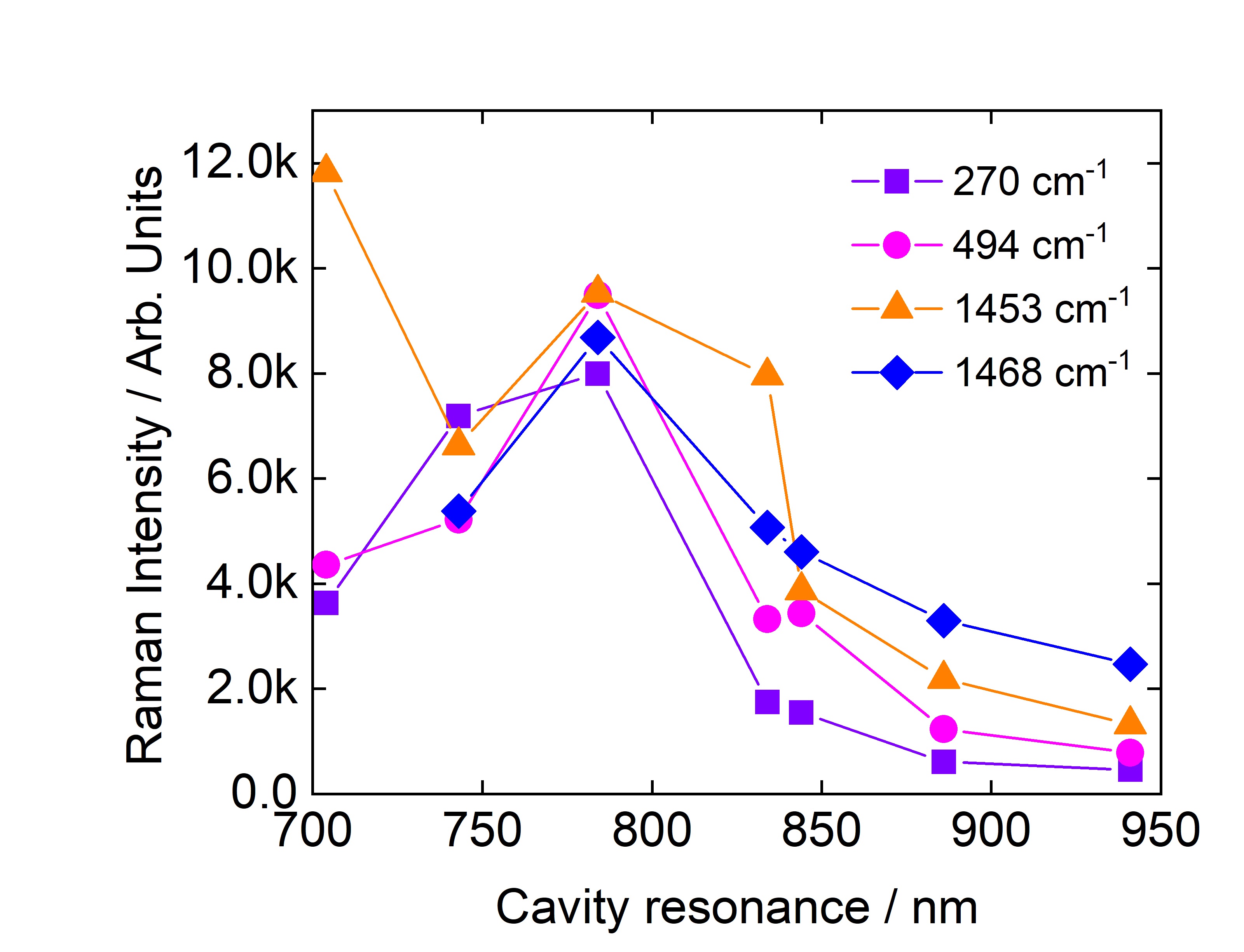

Supplement: Supplementary file 1 [file sensors-20-01470-s001.zip › sensors-711738-supplementary-done/sensors-711738-Supplymentary/images/CERSIntvsCRes.jpg]

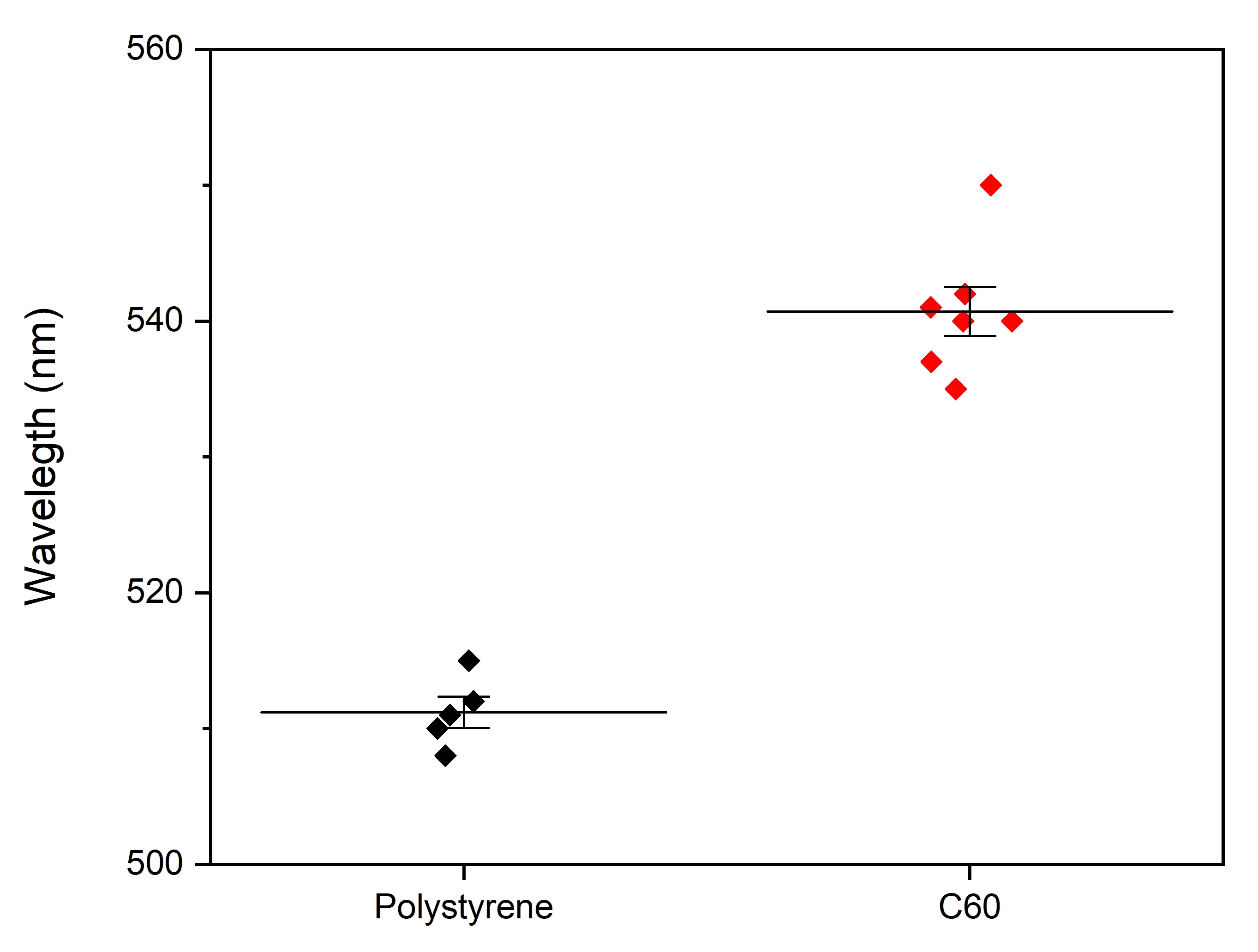

Supplement: Supplementary file 1 [file sensors-20-01470-s001.zip › sensors-711738-supplementary-done/sensors-711738-Supplymentary/images/CompAuAbs.jpg]

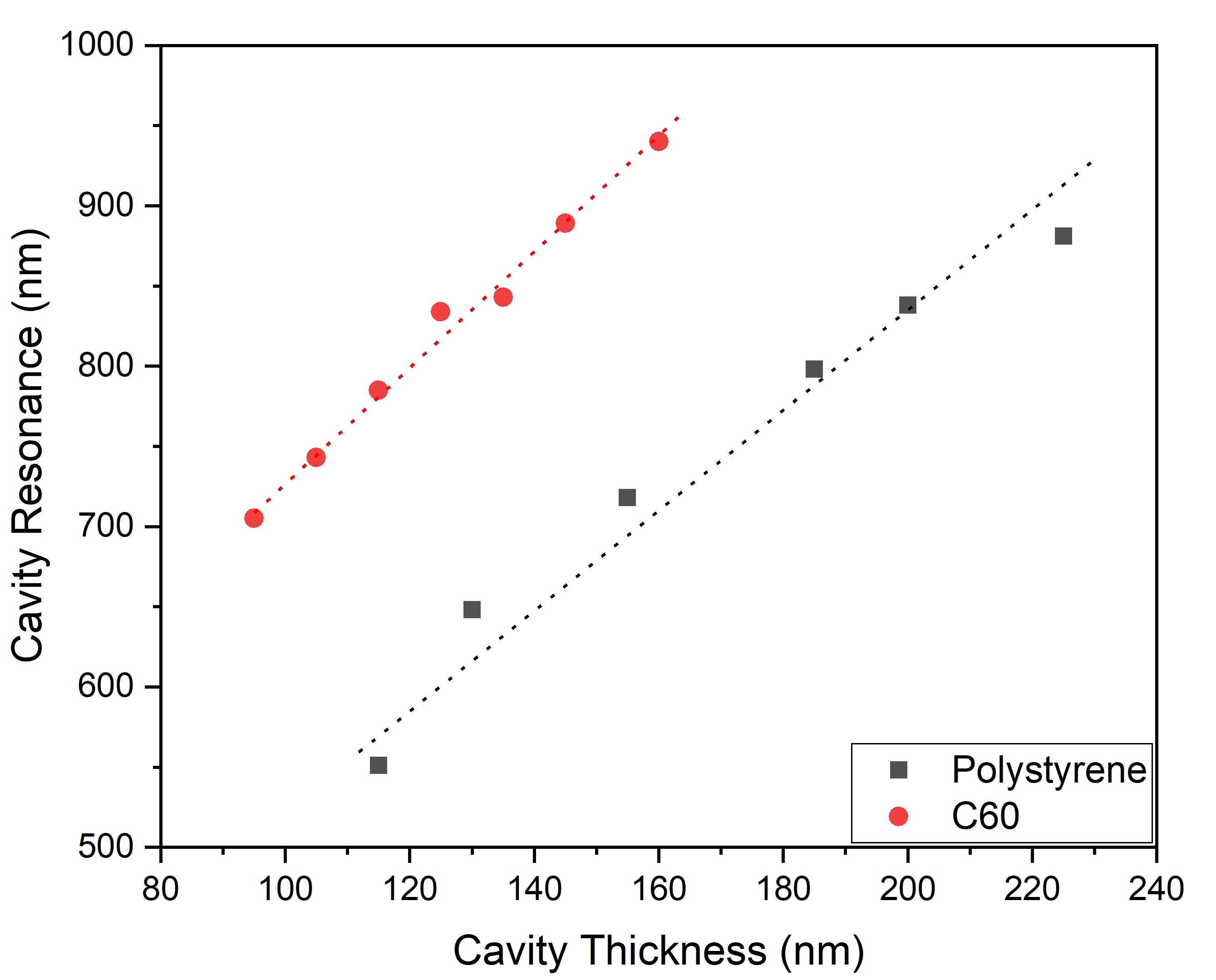

Supplement: Supplementary file 1 [file sensors-20-01470-s001.zip › sensors-711738-supplementary-done/sensors-711738-Supplymentary/images/CompRes.jpg]

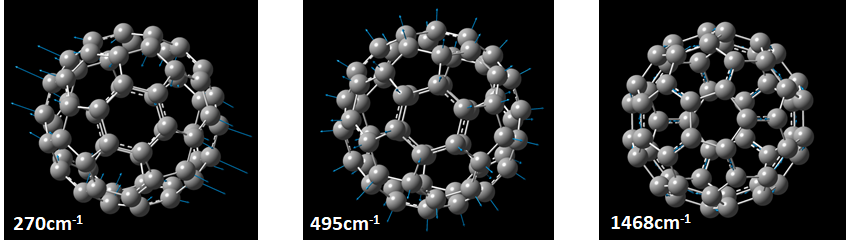

Supplement: Supplementary file 1 [file sensors-20-01470-s001.zip › sensors-711738-supplementary-done/sensors-711738-Supplymentary/images/DFT.png]

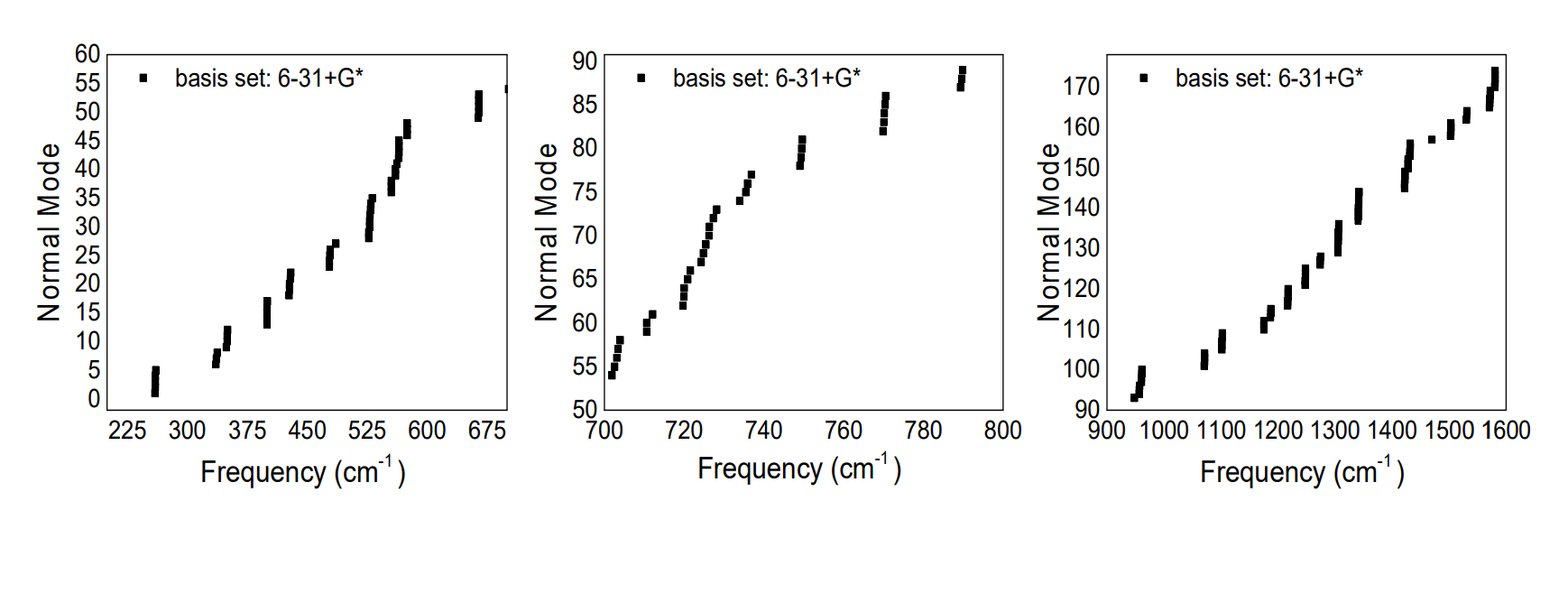

Supplement: Supplementary file 1 [file sensors-20-01470-s001.zip › sensors-711738-supplementary-done/sensors-711738-Supplymentary/images/DFT1.png]

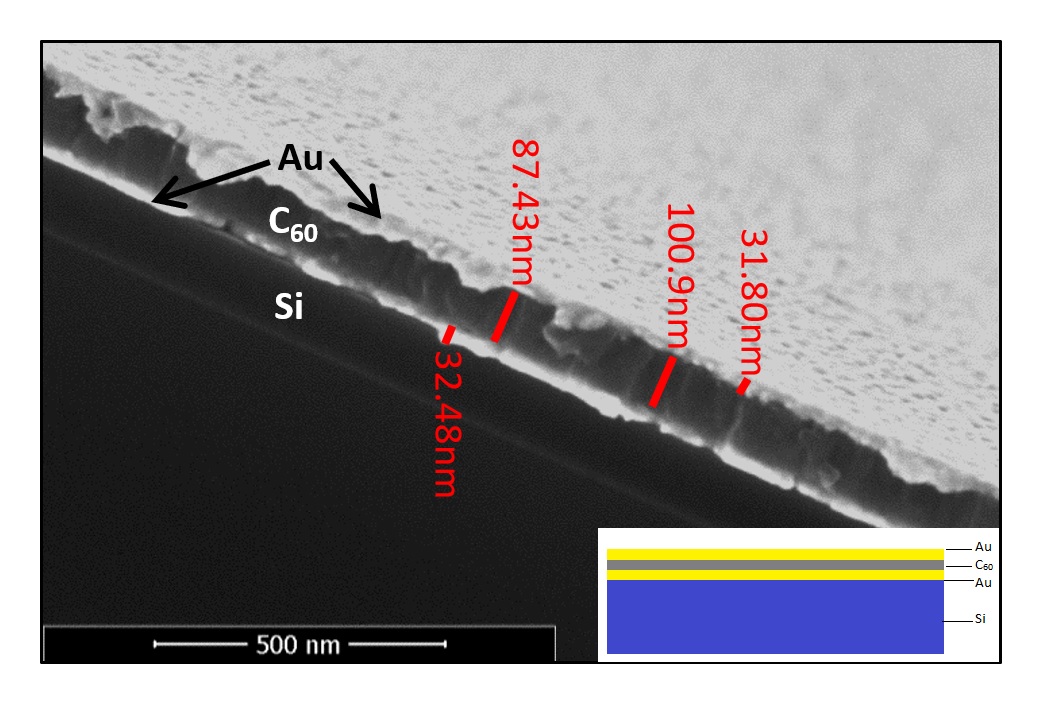

Supplement: Supplementary file 1 [file sensors-20-01470-s001.zip › sensors-711738-supplementary-done/sensors-711738-Supplymentary/images/HRSEM.png]

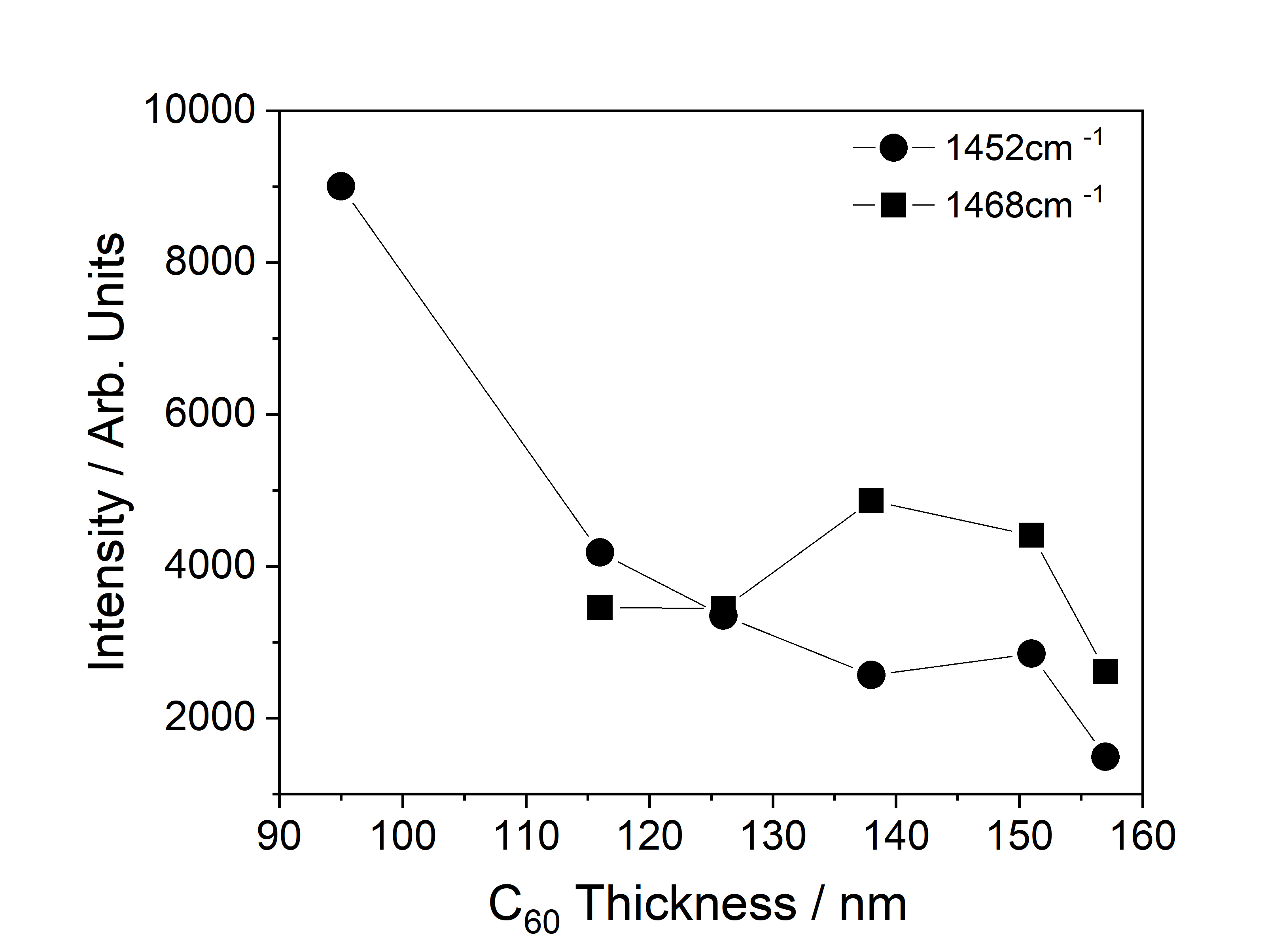

Supplement: Supplementary file 1 [file sensors-20-01470-s001.zip › sensors-711738-supplementary-done/sensors-711738-Supplymentary/images/PeakRes.jpg]

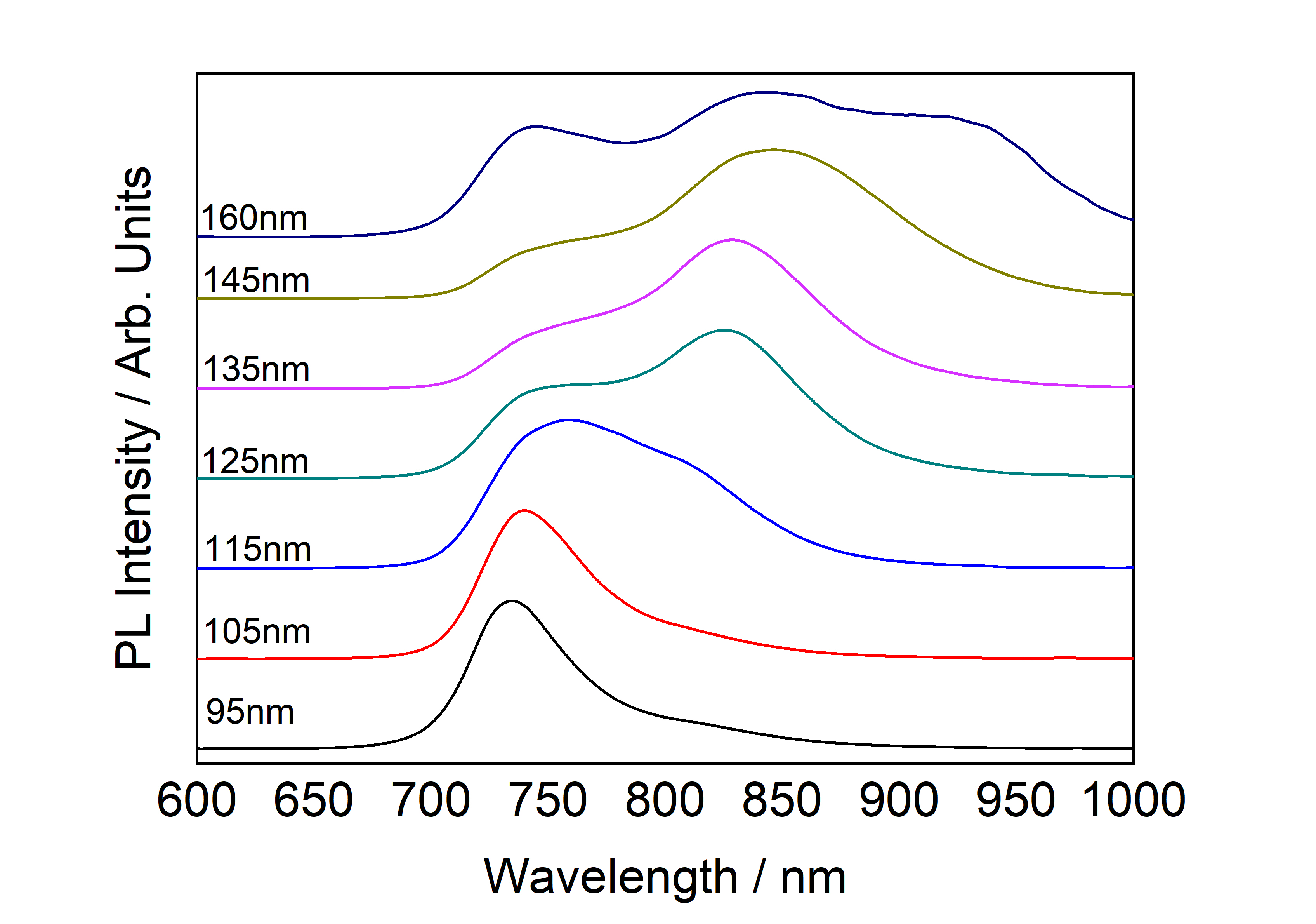

Supplement: Supplementary file 1 [file sensors-20-01470-s001.zip › sensors-711738-supplementary-done/sensors-711738-Supplymentary/images/PL.jpg]

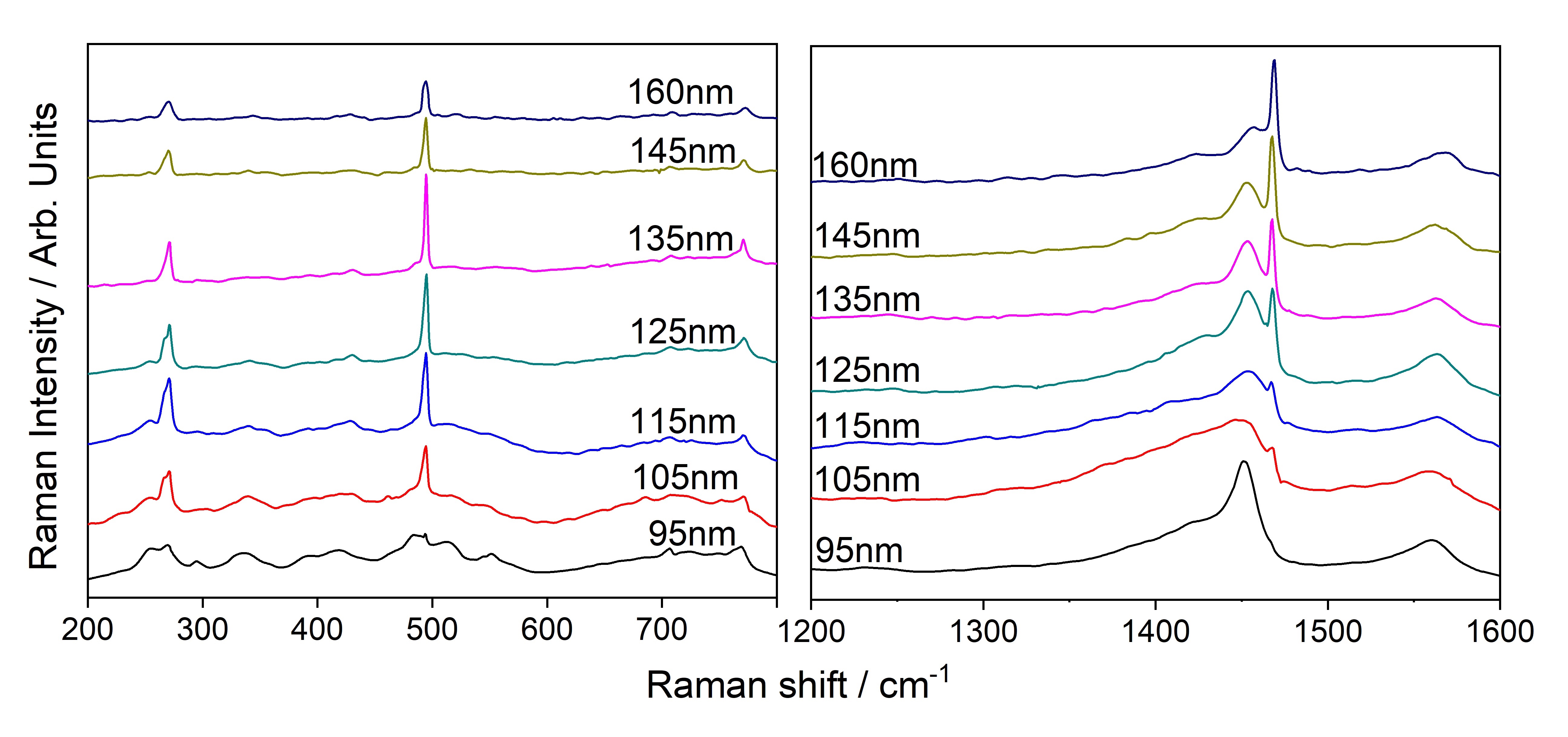

Supplement: Supplementary file 1 [file sensors-20-01470-s001.zip › sensors-711738-supplementary-done/sensors-711738-Supplymentary/images/raman11.jpg]

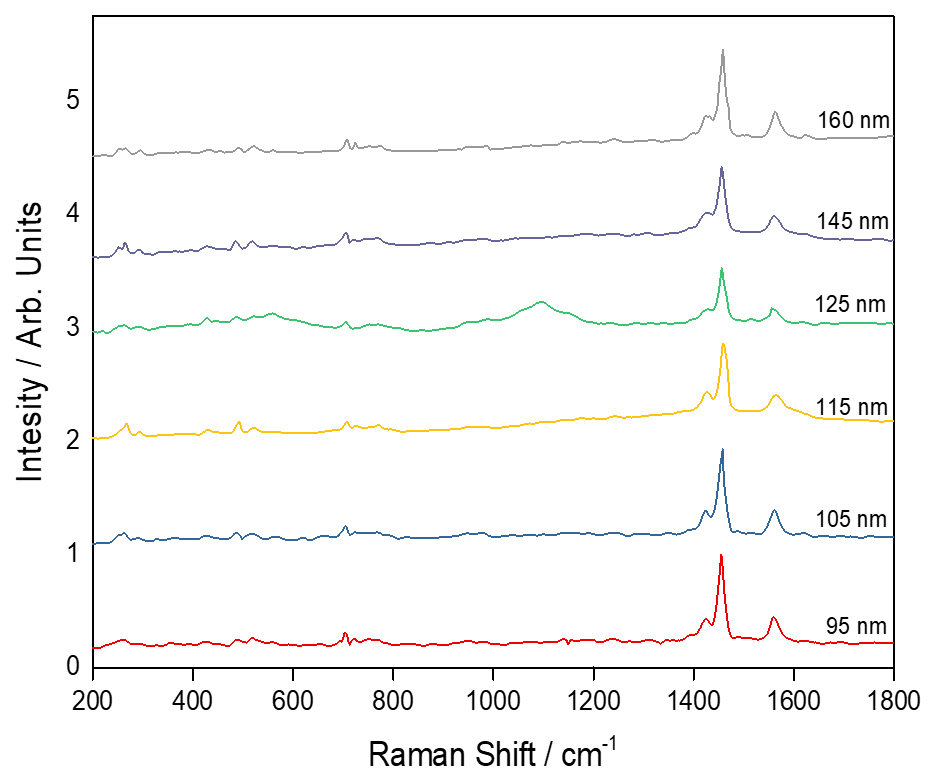

Supplement: Supplementary file 1 [file sensors-20-01470-s001.zip › sensors-711738-supplementary-done/sensors-711738-Supplymentary/images/Raman2.png]

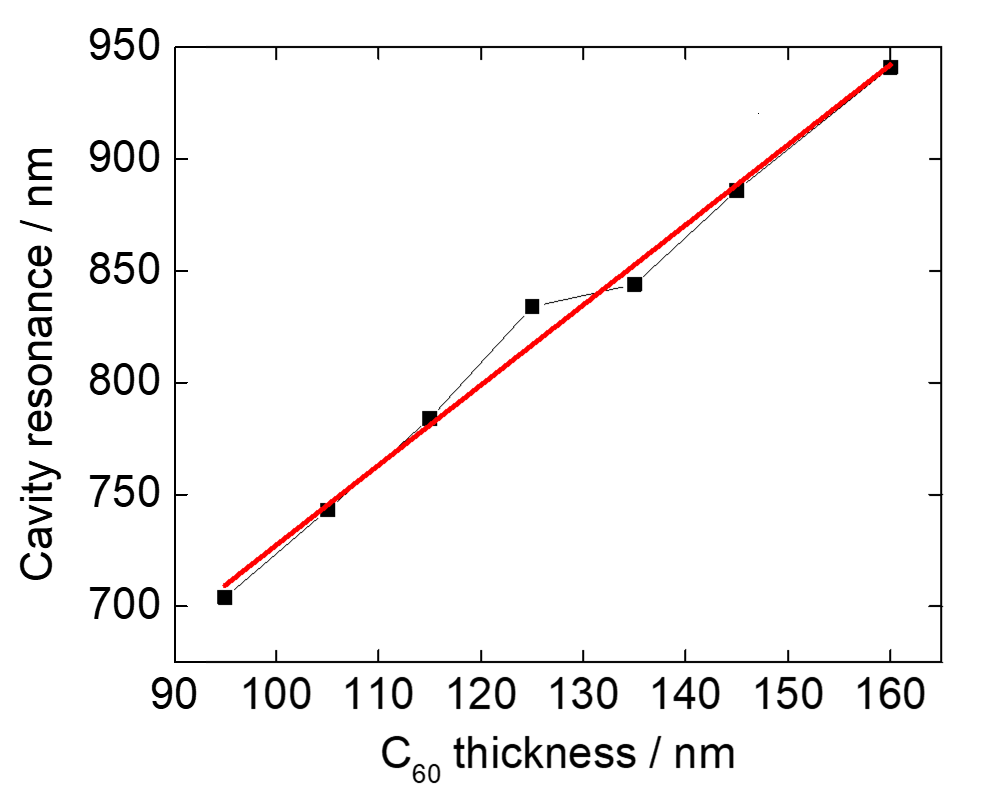

Supplement: Supplementary file 1 [file sensors-20-01470-s001.zip › sensors-711738-supplementary-done/sensors-711738-Supplymentary/images/resonance.png]

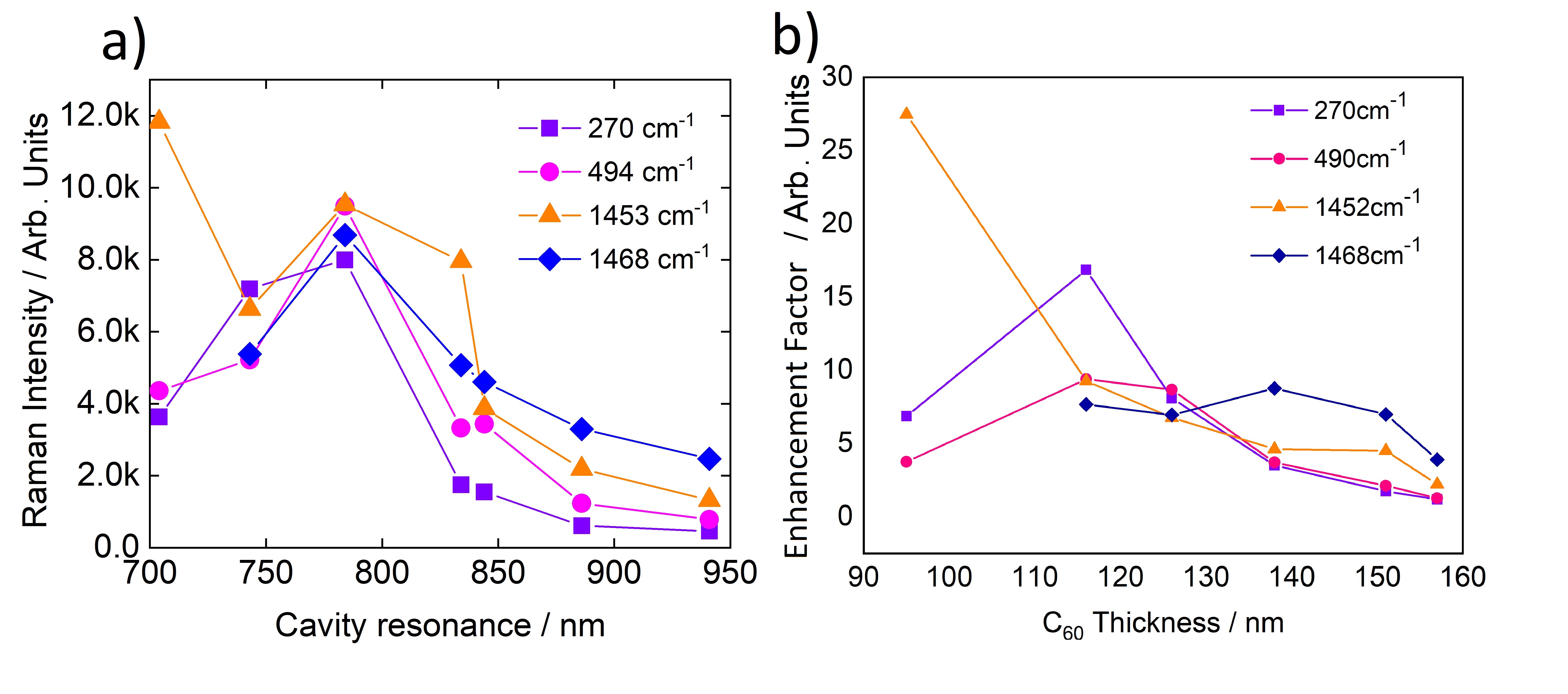

Supplement: Supplementary file 1 [file sensors-20-01470-s001.zip › sensors-711738-supplementary-done/sensors-711738-Supplymentary/images/S3abc.jpg]

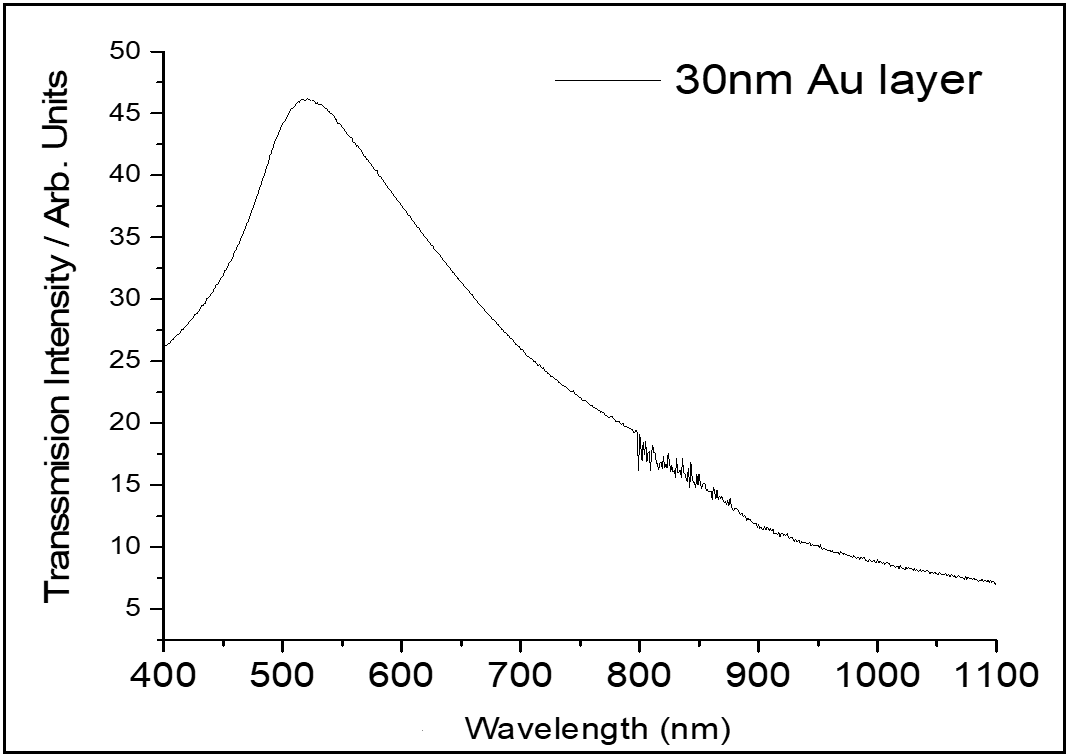

Supplement: Supplementary file 1 [file sensors-20-01470-s001.zip › sensors-711738-supplementary-done/sensors-711738-Supplymentary/images/TransAu.png]

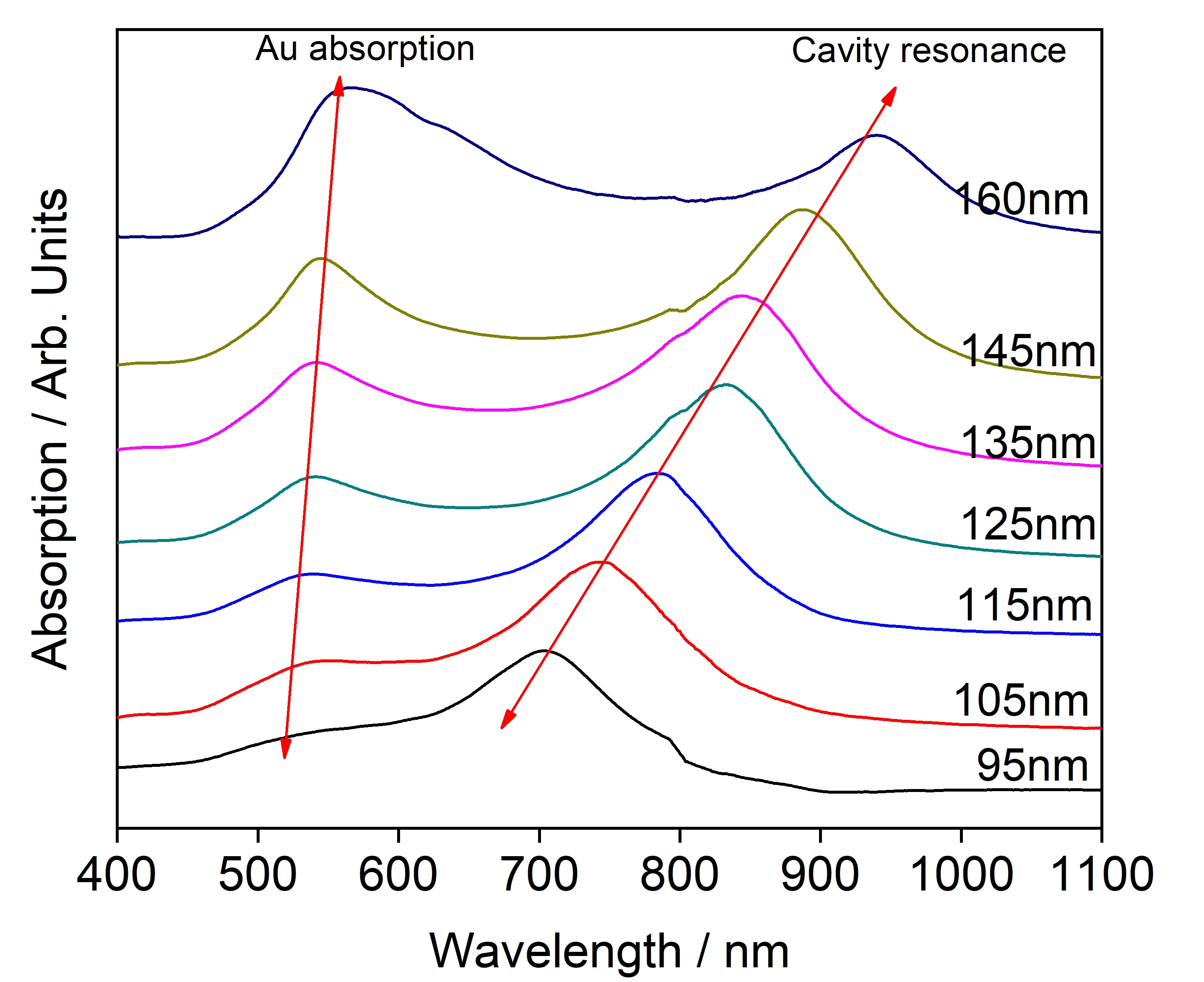

Supplement: Supplementary file 1 [file sensors-20-01470-s001.zip › sensors-711738-supplementary-done/sensors-711738-Supplymentary/images/TransC60.jpg]

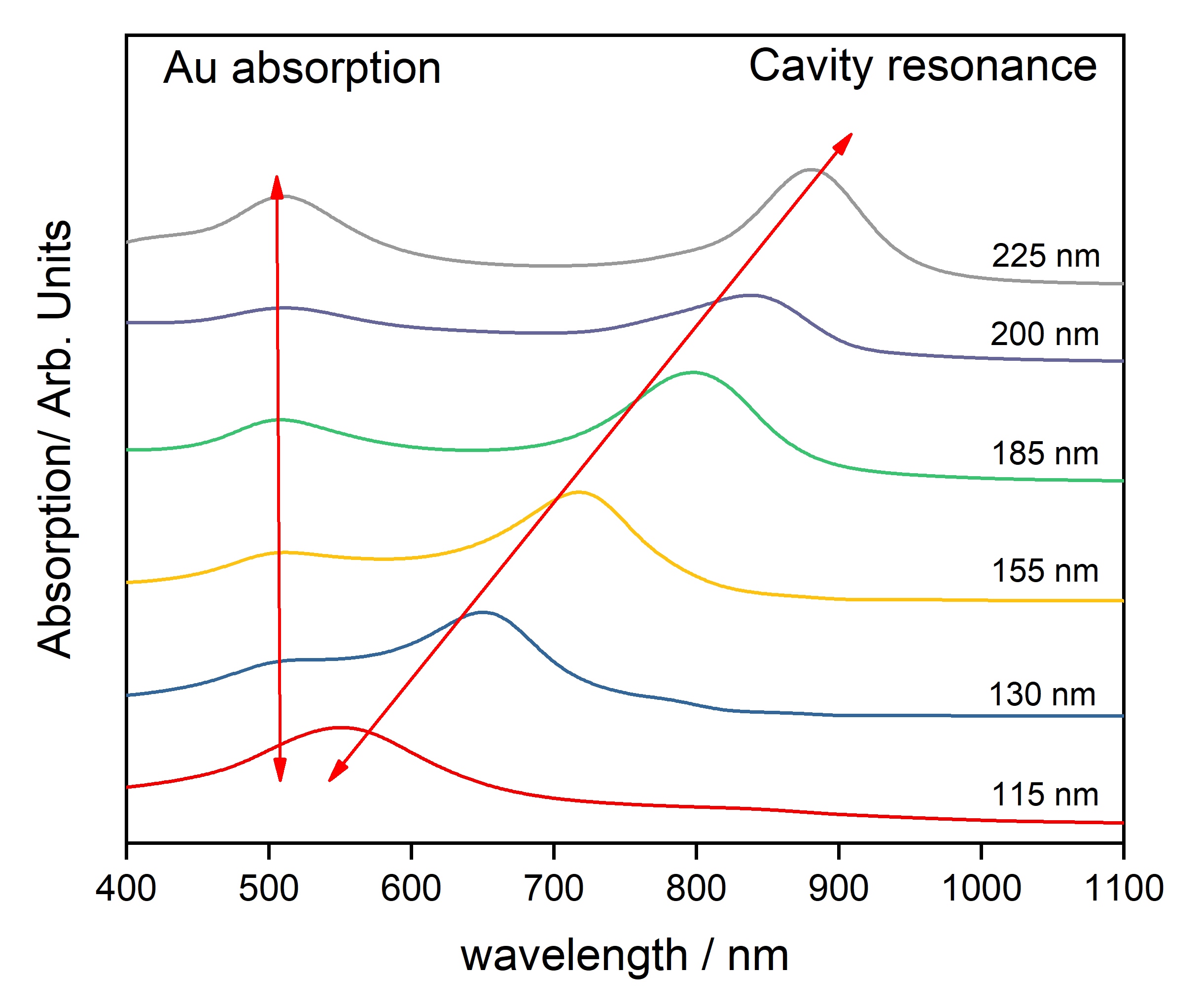

Supplement: Supplementary file 1 [file sensors-20-01470-s001.zip › sensors-711738-supplementary-done/sensors-711738-Supplymentary/images/Transpoly.jpg]

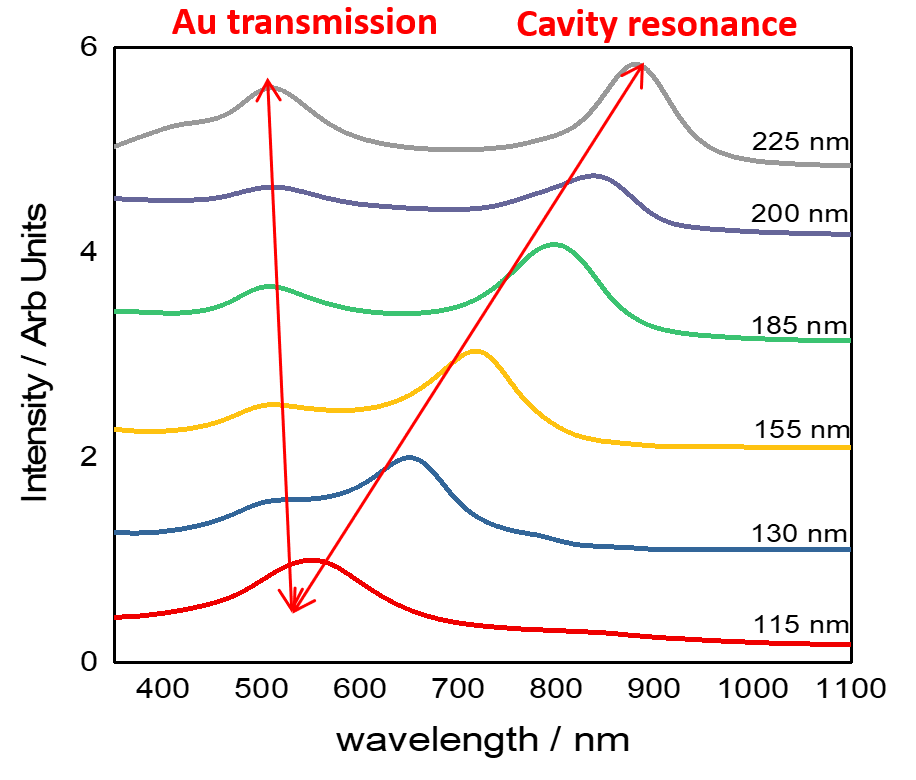

Supplement: Supplementary file 1 [file sensors-20-01470-s001.zip › sensors-711738-supplementary-done/sensors-711738-Supplymentary/images/Transpoly.png]

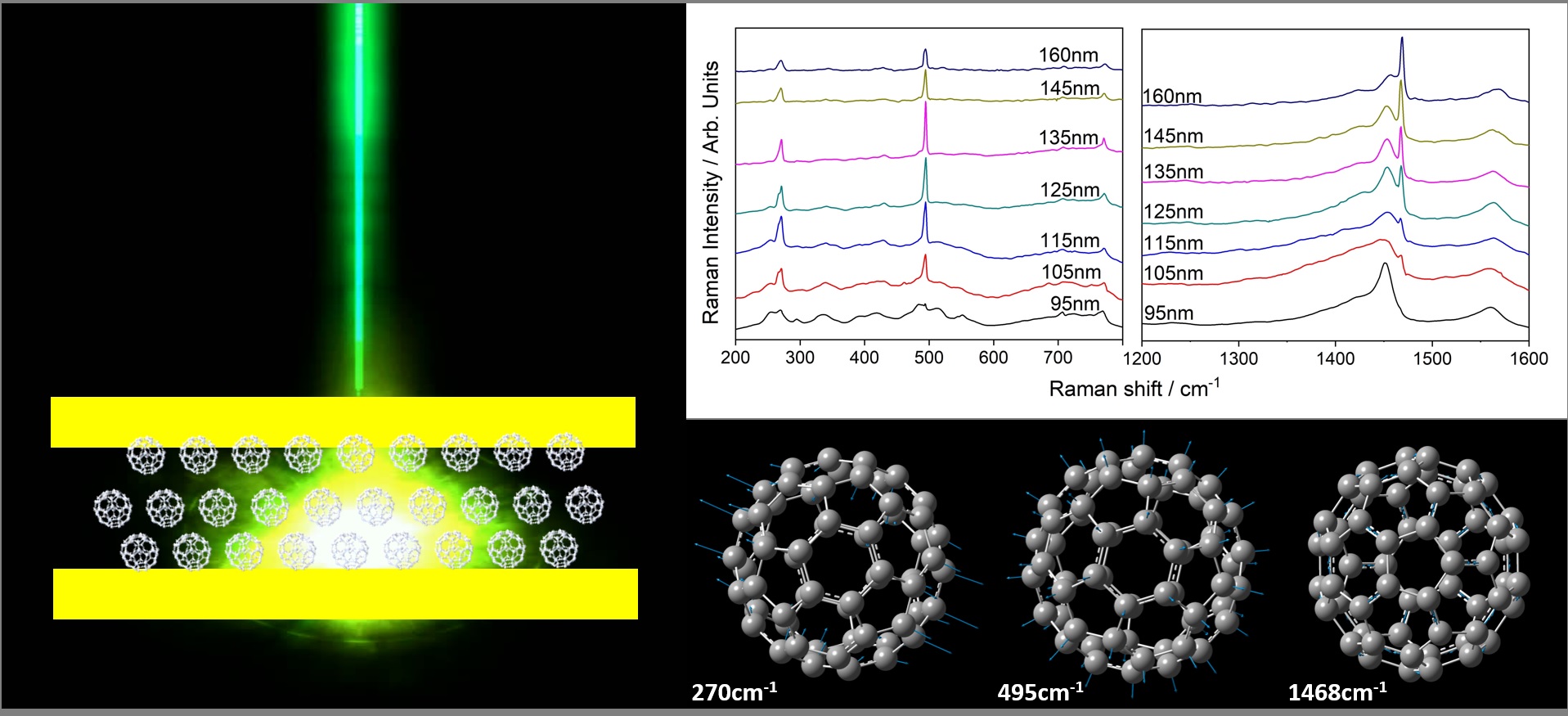

Supplement: Supplementary file 1 [file sensors-20-01470-s001.zip › sensors-711738-supplementary-done/sensors-711738-Supplymentary/TOC.jpg]
